# Pastoral Farming Ethics and Economics–Aligning Grazing Practices and Expectations

**DOI:** 10.3389/fvets.2020.00209

**Published:** 2020-04-22

**Authors:** Mark W. Fisher

**Affiliations:** Ministry for Primary Industries, Wellington, New Zealand

**Keywords:** animal welfare, body condition, shelter, painful husbandry procedures, values, profitability, community engagement

## Introduction

We all generally value animal welfare–what animals experience, how they perform or whether they are being treated with respect–is important to them and to us. However, animal welfare is contested because humans benefit from compromises to animals and we have different expectations borne of different needs, preferences and prejudices: “some we love, some we hate, some we eat” (1). Also contested is what we think and understand is important to animals, and whether the way animals are farmed is in keeping with their nature. In all, a complex subject, but complexity is a feature of humanity, as noted in the development of early civilisations (2).

Few people see the world as exclusively human and most extend concern to animals, plants and the environment, giving rise to different animal-related values, the “rules” and “expectations” we learn from parents and family life, friends, religion, our trades, and professions, the literature, media, the society we live in and its history. Values, then, are not always shared; reasonable people can disagree; and contested ideas can become difficult to describe, subject to considerable political debate and are unlikely to have simple solutions, at best being managed rather than solved. Our common morality holds that we not harm, that we do good, that we are fair and just, and that we respect people's ability to make their own choices (3). The importance of values to human behavior requires that different values are understood, respected and taken into account, even if they cannot be reconciled. And how humans interact with animals is fertile ground for contested practices and expectations.

The aim of this chapter is to provide a commentary on some of the contested aspects of pastoral farming and how we go about dealing with them. The purpose is to reveal the complexity of the subject and how this gives rise to different views or perspectives informed by different values, and how we engage and evaluate those views.

## The Challenges of Pastoral Farming

Farming is at the nexus of two worlds, the physical or biological and the social or societal, farming arguably subordinate to both (4). Consequently, the potential challenges can be varied, from dealing with the vagaries of climate to the expectations of people far removed from the land. Most pastoral farmed animals, especially those in extensive environments, have some of the attributes of free-living or wild animals. Although having choice of diet and considerable freedom of movement and behavior, they are under some degree of human management (5–7). For example, social and kin structure may be distorted by culling and grouping, movement may be limited or prevented, food and nutrients are often less varied in composition, parental care of young may be curtailed by weaning, and animals are usually less subject to predation and natural selection but increased artificial selection is likely. Furthermore, changes to pastures, animals and management such as supplementary feeding and artificial breeding are commonly used to minimize ecological constraints and improve animal and farm productivity and efficiency (8, 9). In such a complex physical and social environment, there are potentially many challenges to animal welfare, including, for example, those related to body condition, shelter, exposure to mud, and painful husbandry procedures. These examples are used to highlight some of the different perspectives contributing to animal welfare, perspectives that different people may value differently.

### Body Condition

Animals have evolved homeostatic mechanisms to overcome nutritional variation when energy demand exceeds availability, e.g., in winter, during pregnancy, and lactation, or when unable to forage. Body condition score, an accepted measure of energy reserves, generally reflects better access to resources, individuals exhibiting enhanced survival (10–12), growth, reproduction, lactation, and health [e.g., (13, 14)]. However, the relationship is not simple. Dairy cows in higher condition at calving are at greater risk of metabolic disorders (e.g., ketosis, milk fever), while those in lower condition are more likely to have difficulty getting pregnant again. Body condition is, then, an important management tool for optimizing flock or herd health and productivity. For example, body condition can be reflected in ovulation and lambing rates (15), lamb birth and growth rates (16), calf birth weights and earlier returns to breeding (17).

As well as inadequate feeding, loss of condition can reflect underlying disease or parasitism, and is sometimes more prevalent at the end of an economic or productive life. It can also be the result of neglect or failure to care for animals, sometimes the result of people in difficult financial and personal circumstances, such as during droughts, or ill-health, and challenging relationships [see (18)]. Furthermore, maintaining animals in good condition may not just reflect a lack of food, but changing genetics (e.g., selection for production can be at the expense of body condition) and management (e.g., a lack of skills in managing pasture, perhaps exacerbated by farming to generalized recipes rather than having the skills to adapt to novel and changing situations).

Not surprisingly, animal welfare regulatory codes place importance on body condition. Typically, they require that when animals are thin, emaciated or very thin, urgent remedial action is taken to improve condition, or the animal humanely destroyed. Therefore, few farm animals tend to routinely be in such poor condition. However, as has been noted (10, 14) neither the animal welfare decision process, nor the information on which recommendations for optimal body condition are based, are clear. The standards seemingly rely on common sense–emaciated animals represent poor welfare, or poor productivity. However, there are limitations in extrapolating from body condition to animal welfare. For example, at low condition score, the weight of fat in dairy cows may be overestimated (19). Even within a more normal range of scores, body condition may have its limitations. After monitoring milk production, health, and udder and uterine health, Roche et al. (20) concluded that body condition score (at least between 3.5 and 5.5 on a scale of 1–10) “is not a sufficiently sensitive measure to be reflective of cow welfare” in early lactation, despite it appearing optimal for production, reproductive performance and general health. For example, based on measures of liver and immune function, the authors suggested that body condition peripartum and early lactation did not affect cows' abilities mount an inflammatory response.

As well as the understanding of body condition being dominated by measures of animal productivity, further reflection on the relationship with animal welfare reveals a number of other limitations. Firstly, as well as being diagnostic of nutrition, body condition is both a dynamic state and a subjective assessment. Animals at a particular condition score could be maintaining, gaining or loosing condition with different implications for what they feel (e.g., hungry) and are likely to experience (e.g., metabolic diseases), as well as reflect underlying conditions such as ill-health, parasitism, or advanced age, and their seasonal physiology. While it is reasonable to assume that feed restricted animals might be hungry, body condition may not necessarily reflect, or can be taken as a measure of hunger (21). For example, there is natural variation in body condition between individuals and breeds, as well as species. The physiology of some animals may see them lay down fat but then lose their appetite seasonally, e.g., rutting stags. Similarly, an analysis of death rates amongst sheep being exported by sea from Australia suggested that sheep in the fat deposition stage were less able to adjust to feed deprivation (22). It should not be assumed that animals in good condition do not experience hunger, especially when they have been bred to be highly productive. Some sows and breeder meat chickens display extreme examples of what Rauw et al. (23) have suggested is altered, perhaps pathological, hypothalamic mechanisms regulating appetite.

While body condition can be an indicator of hunger, and thus welfare, it is more precisely an indicator of past access to nutritional resources and thus an ability to deal with the constraints of the future environment, be it climate or a need for veterinary care. As such, it serves to illustrate the difficulties in relating how an animal performs with what emotions it may be experiencing, two of the predominant understanding of animal welfare. Nevertheless, body condition is an iceberg indicator, i.e., a key indicator of overall welfare inferring that the animal's care is of high quality and its welfare good (24).

### The Provision of Shelter for Pastoral Animals

**Figure 1:**
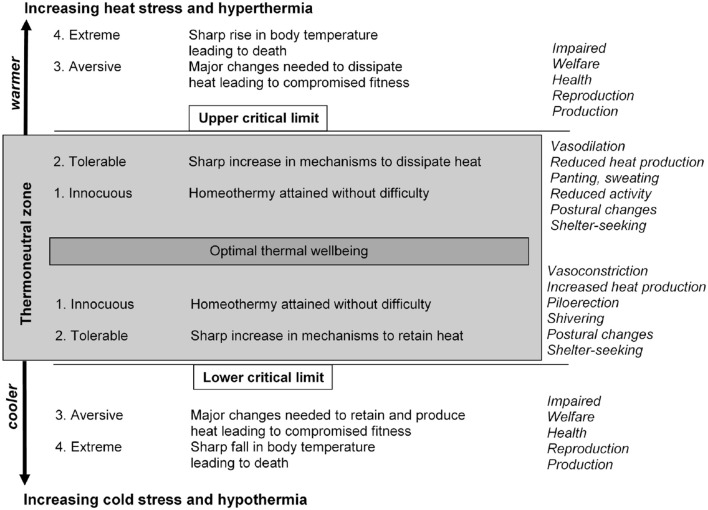
The responses of animals, and the consequences, to thermal challenges evoked by exposure to increasing heat and cold stresses [from (25)].

Pastoral animals experience and usually successfully adapt to a range of climatic factors, whether they are daily and seasonal or extreme and adverse. Adaptation to thermal challenges involves a range of physiological and behavioral systems (Figure 1), including shelter seeking behavior, shelter being a resource animals need to ensure their comfort, productivity and survival.

There is a seemingly endless range of possibilities enabling animals to lessen the impacts of adverse weather–contours in the land, hedgerows, trees, gullies, flaxes, vegetation clumps, tussocks, rocks, woolsheds, rushes, etc. The importance of shelter is noted in the internationally recognized “five freedoms.” Freedom from discomfort by providing a suitable environment including shelter and a comfortable resting area (26) addresses, like the provision of food and water, a fundamental need. However, although there are many directives and expectations for the provision of shelter, it remains an example of what Dwyer et al. (27) describe as “stubbornly unchanging.” The accumulation of knowledge is sometimes not having a significant impact on the circumstances animals encounter (28).

Given that shelter is important and valued by animals, farmers and the wider community [e.g., (28)], why is it an issue? In a survey (29) barriers to the greater provision of shelter for pastoral animals included resources (time, money, return on investments, etc.), the negative impacts on farm productivity (e.g., the removal of shelterbelts to enable the use large, center-pivot irrigators), inadequate knowledge of means of providing shelter and their success, and the fact that standards are difficult to enforce requiring proof of animal suffering and with a lack of consequences for not providing shelter. Furthermore, many farms appear to provide adequate shelter; others have active plans to provide more shelter; some have other priorities; or are more resistant requiring exposure to the consequences including legal enforcement. In addressing shelter, it may be necessary to acknowledge these factors and aim to achieve a balance between regulation and enforcement and incentives and encouragement.

### Exposure to Excessive Mud

Winter brings many challenges, most notably meeting animals' nutritional needs (if well-fed, livestock can usually tolerate variations in the weather). Mud is the inevitable outcome of slow pasture growth in winter, exacerbated by rain, and/or intensive land use, and high stocking densities. It can be uncomfortable, cold and wet. Furthermore, images of miserable-looking animals, deep in mud, has led to public criticism and expectations for improvement (30). Storms, stocking densities, animal preferences, pastures, aspect, soil types, management needs and all the other things that make farming both rewarding and challenging, mean that mud is sometimes inevitable. While feedlots, feeding pads and crop utilization are pragmatic examples of ensuring animals have adequate nutrition over winter, as well as reducing pasture damage and improving feed utilization, they mean animals are concentrated on small hard-surfaced areas. Advances in crop breeding producing markedly increased dry matter per hectare can mean animals live on smaller areas for longer periods, often resulting in mud. On feedlots, energy requirements increase if animals are wet and muddy, especially if not sheltered from the wind, thus mud can also reduce animal performance. Excess energy requirements depend on mud depth, temperature, portion of animal affected, and wind. Liveweight gains can be reduced by 35% in dirt pens in muddy conditions, and cattle need about 25% more feed to produce the same gains (31).

Excessive or prolonged exposure to mud, especially very wet mud, can potentially impact on animals' needs as follows:

- Proper and sufficient food and water–feed can become contaminated and more difficult to access leading to animals “giving up” and becoming hungry and losing condition, leading to increased risk of metabolic diseases, physical weakness and an inability to stand.
- Adequate shelter–if the site is exposed to adverse weather, especially extremes, with animals unable to seek shelter, animals are prone to discomfort, shivering, hypothermia, and death.
- Opportunity to display normal patterns of behavior–an inability to rest properly because of a preference for dry comfortable resting areas, may result in reduced lying, tiredness, reluctance to move greater distances, inability to move, isolated from mob; possibly increased risk of infection, and typical physiological indicators of stress (more so than moderate feed restriction).
- Protection from, and rapid diagnosis of, any significant injury or disease–mud can increase susceptibility to lameness, mastitis, dermatitis, hypothermia, and metabolic diseases.
- Animals in mud could then, experience discomfort, chilling, skin irritation and pain, weakness, exhaustion, frustration, and depression.

**Figure 2:**
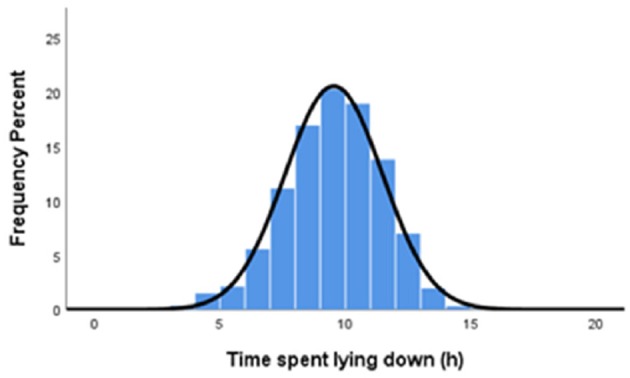
The distribution of lying time (mean 9.5 h) in pasture-farmed dairy cows (1948 cow-days across 10 farms) in large (> 500 cows kept as a single group) herds in Australia (32).

One of the less well-appreciated animal behavioral impacts of mud is on the ability of animals to rest. Dairy cows, for example, may spend, on average, 10–12 h per day lying down although there is much variation between individuals (Figure 2). However, lying time is significantly reduced when the animals are exposed to muddy conditions, as little as 3–6 h during the first 24 h. Chen et al. (33) concluded that “muddy conditions, even in the absence of wind or rain, are aversive to cattle and have negative implications for their welfare.”

An animal's preferences for lying is dependent on its environment. A dry and comfortable surface, such as pasture, woodchips, or sawdust, is strongly preferred by dairy cows (34). On a wood-chip standoff pad, lying time reduced from an average of 11.6 h a day to 5.6 h over 5 weeks when bedding was not refreshed (35). If faced with choosing between feeding and lying, dairy cows prioritize lying, and depriving animals of the opportunity to lie down, for example when surfaces are wet and dirty, impairs welfare, evident in in altered pituitary-adrenal function indicative of stress (36, 37) and immunosuppression with an increased the risk of infection (33). The importance of lying was seen in tired cows, those from wet, uncomfortable standoff surfaces, preferring to lie down when they might have normally been expected to graze. The altered lying pattern of a herd, more cows lying down sooner, and for longer, on returning to pasture, may be a simple and practical indicator of inadequate resting opportunities on standoff surfaces (35). Interestingly, rumination and resting appear inextricably linked. For example, sheep given finally chopped feed that did not require ruminating became tired and irritable. The addition of hay to their diet enabled rumination and the disappearance of distress and exhaustion (38).

The risks of excessive mud appear to be minimized by providing fresh bedding, environmental buffers (e.g., windbreaks, mounds, shelter) and access to space with dry, comfortable resting areas. Cow cleanliness is becoming an accepted indicator of animal welfare in farm assurance programmes and dirty cows (e.g., flanks, hind limbs, and udders) a measure of an unsuitable environment.

### Painful Husbandry Procedures

Fences notwithstanding, one of the features of pastoral animals is that they more likely to be able to behave naturally compared with more confined farm animals. However, and like many of their more intensively farmed counterparts, livestock are subject to painful husbandry procedures in order to enhance animal health and welfare, facilitate husbandry and management, enhance animal products, or reduce the safety risks to humans. Common examples include castration, tail-docking, and disbudding and dehorning. Many of these procedures can cause anxiety, fear, discomfort, pain, or distress associated with mustering, handling and restraint, and acute and chronic pain resulting from the physical interference of sensitive tissues. While many of these impacts have been well-documented and have contributed to greater use of pain relief at the time of the procedure, the possibility of modified behavior resulting from the procedure is less well-known. One example is the practice of tail docking dairy cows to supposedly improve hygiene, in part because of the reduced inability of the animal to swat flies and to communicate its emotional state. However, perhaps the best example of a husbandry procedure designed specifically to thwart behavior, is nose ringing in in grazing pigs. Although not a ruminant, the example is insightful because of the thwarted behavior being the more significant compromise to the animal than other husbandry procedures where the procedure, usually performed without pain relief, is arguably the more significant compromise.

Rooting is the means by which pigs explore and search for, locate and harvest food. The insertion of a ring, clip or wire through either the nasal septum separating the nostrils, or the upper, outer part of the snout, is a common means of preventing pigs from undertaking the behavior, principally to reduce pasture damage, and perhaps lessen soil erosion, nutrient leaching, and internal parasites. Nose rings reduce the time a pig spend rooting (20–30% of waking hours in semi-natural conditions) and result in significantly better grass cover (39).

The “extreme vocalizations” at the time of insertion suggest nose-ringing is painful. It is probably also painful for a period after the procedure since some behaviors take time to return toward normal levels. Typically, pain relief is not provided. However, it is the long-term or chronic effects which are arguably more significant. Firstly, the ring is effective in reducing rooting presumably because it is uncomfortable or painful to root with a nose-ring. Several behaviors are affected with differences in grazing, sniffing, standing inactive, pawing/scraping the ground, and chewing straw, as well as rooting, evident with different rings or clips (40). Secondly, a natural behavior thought to be important to the animal is thwarted, resulting in a degree of suffering although signs of frustration may not always be evident (41). Pigs continue to root in intensive systems even when fed *ad libitum* and housed on wire-mesh or concrete floors, situations where they perhaps have no need for food, and certainly no prospect of digging. This suggests rooting is an important natural or normal behavior, and preventing it is likely to lead to frustration and altered behaviors. Finally, the rooting action is also part of digging wallows, nest building, physical aggression and exploring. Ringing reduces rooting in wallows and in straw, and affects grazing and palpating the ground for nuts, stone-chewing and increases the amount of time spent standing but otherwise inactive. Ringed animals are at a disadvantage when feeding and may need to be kept separately from un-ringed animals to enable the former to obtain their fair share of feed (42).

There have been attempts to provide opportunities to satisfy or divert pigs' urges to root. For example, providing other things to do, or a more satisfying diet; sacrificing rooting areas or provision of root crops; or providing earth- or peat-filled rooting troughs in intensively housed pigs. While the provision of root crops did not appear to prevent rooting in any significant way, the provision of rooting trays resulted in less abnormal behavior, such as belly-nosing or ear and tail biting in intensively housed weaners.

Clearly there are compromises to animals undertaken for human benefit, compromises that different people have different views on. Furthermore, alleviating those compromises inevitably comes at a cost. Typically, we appeal to ethics and economics for some sort of guidance, or to justify compromises are reasonable and necessary, in other words what costs should be borne by the animal or the farmer. The following section is a general introduction from the perspective the author's interest in animal welfare, as well as science and farming.

## Ethics and Economics

Any introduction to ethics should emphasize two things–it is complex, and yet it is something we all do. Ethics' complexity is one of its benefits. We and the world we live in, are complex–simplifying it doesn't always work.

Morality has its roots in co-operation between social beings over resources–the land, animals and people, and how we apportion them as well as take responsibility when things go wrong. The terms ethics and morals are often used interchangeably but the former can be understood as the study of the latter, the thinking or “reasoning” behind beliefs of right and wrong actions. There are many different reasons and theories–like different scientific disciplines, e.g., animal behavior, immunology, and reproduction–each providing different and important insights, but also each having their own limitations.

The most well-known theory, consequentialism, is based on whether the likely consequences of an action will have benefits outweighing the harms. For instance, humans have benefited from the milk production of dairy cows, both as a source of food and of industry and commerce. These benefits are taken to outweigh the harms associated with the removal of the calf from the cow at birth, and either their artificial rearing or imminent slaughter. These practices are common to many modern dairy systems. However, relying exclusively on an ethic based on the benefits outweighing the harms is problematic. It does not mean all harms to animals are justified because of the benefits to humans. As both individuals, and as a society, we accept that there are some things we cannot do to animals, no matter what the benefits are. For example, as Rollin (43) asks farmers, would you do anything at all to increase profits and production, such as “torture a cow's eyes with hot needles if it increased milk production?” Rights theories set a limit to actions, regardless of the benefits. This ensures, depending on the circumstances, that there is a limit to animal compromises.

Rights are a social device that makes it easier for people to live with each other by providing a protection or constraint on treatment. Western animal rights theory appears to have evolved from eighteenth century reaction to humans apparently having no obligation to animals or to their treatment. Not surprisingly, the movement resulted in almost complete consensus in the need for the speedy killing of animals when slaughtering, or in eradicating vermin, and in repudiating cruelty to animals (44). This view of rights is reinforced by the idea that animal welfare and animal rights are, despite common perceptions, essentially similar in aims (45)–animals have entitlements or rights to adequate food and shelter etc., which humans have a duty to provide (both for the animals sake and because it makes us better human beings). While the term “animal rights” can, and commonly does, refer to any call for the fair treatment and protection of animals, the more revolutionary rights theories maintain that, because animals are the subjects of a life, they should not be used for farming, in animal research and testing, or even kept as pets (46). While it is understandable, then, that animal rights is often dismissed, especially when associated with revolutionary implications and illegal activities, such a stance does not reflect the complexity and common understanding of the term. On the other hand, common understanding of animal rights may preclude its use amongst some people, even where consideration might result in more equitable use and treatment of animals.

Another ethical theory is pragmatism. It not only considers the consequences of an action, but also emphasizes the legitimate and necessary role that emotions and sympathy play in moral reflection and choice. It is, for example, difficult not to feel something for dairy cows, with whom you've become associated with over many years, being loaded for slaughter at the end of their productive lives. Thus, some ethical theories are impoverished in not realizing the special weight of relationships usually inherent in husbandry and caring (47) and this is most evident in care-based reasoning, especially actions that are informed by an intimate understanding of the circumstances, in the skills of stockmanship and animal care based on a respect for the essence of the animal (48). Individuals commonly draw on tradition and experience and a willingness to learn, personal qualities of empathy and patience, and an understanding of the balance between the expectations of people and the needs of animals in different systems. The attributes of good stockmanship include:

- being able to draw on a lifetime, or intergenerational catalog of practical personal experiences with animals and farming such that actions become second nature, where feel and experience are valued as much as specialized knowledge and measures;
- having personal qualities of patience, empathy and other traits or attitudes considered necessary for working with animals, their impacts having been demonstrated scientifically in modern pig, poultry and dairy cattle farming systems [e.g., (49)];
- an understanding of the constraints and opportunities afforded by the farm environment including the terrain, the climate and the flora and fauna, experience with animals aligned with that of the land and the weather; and
- knowledge of the normal behavior of animals and, being observant, having the ability to recognize and deal with abnormal behaviors.

Much of this knowledge is ineffable and should not be underestimated in the calls for formal training and proof of competency, invaluable as they are in complementing such understanding.

Ethical theories, then, provide different insights for people and one understanding of ethics is that it is the systematic examination of moral issues in the public sphere. The broad view, representing the culmination of a long tradition of moral reflection, as well as expressing the common view of most members of society, is that the use of animals is permissible as long as it is justified and humane (50), respecting the following three principles:

- Harms of a certain degree and kind should not be inflicted on an animal, regardless of any benefits (e.g., mulesing, the surgical removal of skin around breech of lambs to reduce the risk of flystrike, or the use of blunt-force trauma to kill unwanted dairy calves, except in emergencies, are prohibited in New Zealand).
- Any harm (e.g., physical pain, loneliness, degrading use) to an animal must be justified by ensuring the good realistically expected from the harm, outweighs the harm inflicted (e.g., disbudding or dehorning cattle to reduce the risks to human and other animal safety).
- Any harm which is justified, should be minimized as far as is reasonable possible (e.g., undertaking painful husbandry procedures on young animals to reduce the amount of tissue involved, and providing pain relief).

The third of these principles, minimizing the harms, essentially describes good standards of practice, often justified by or drawn from science [e.g., (51)] and good husbandry and encoded in codes of welfare. New Zealand's *Sheep and Beef Cattle, Dairy Cattle*, and *Painful Husbandry Procedures* codes of welfare (52–54) describe, for example, stockmanship and animal handling; the provision of food, water and shelter; opportunities for animals to behave normally; addressing health, injuries and disease; and husbandry practices from selection and breeding to animal identification, humane destruction, and minimizing pain and distress.

Although codes of animal welfare have a regulatory role, failure to meet a minimum standard can be used as evidence to support a prosecution whilst equalling or exceeding such a standard can be used as a defense, codes typically have a number of uses and purposes. For example, they also articulate the aspirations of society; raise awareness by drawing attention to issues; give the public an idea of what to expect; and are self-promoting, the standards defining good animal use in a particular industry or country serving to differentiate them from those ascribing to other, especially lesser, standards.

Although there are many ethical insights reflecting common morality and given expression in codes of welfare, arguably one of the more significant is the economic benefits to humans justifying the compromises to animals.

Like animal welfare (55), the term economics has numerous understandings. At one level, it has a business focus, the need to cover expenses and maintain a profit. This is one of the goals of agriculture, along with producing safe and affordable food that is produced fairly and without harming the environment, animals or people. Maintaining profitability is a challenge for any business as decreasing returns and increasing costs squeeze profits. This leads, in many cases, to larger operations benefiting from economies of scale evident in larger farms, flock and herd sizes. And higher animal welfare standards often come at a greater financial cost to the farm system [e.g., see (56–58)]. Profitability is challenging when viewed against the long-term trend of an increasing marketing share of the consumer's spending on food at the expense of the farmers' share (59).

Animal welfare is also an economic concept at another level: the trade-offs we make between our preferences for food and the opportunities for commerce in producing it, vs. our discomfort with whatever animals may experience in the process. Varying social expectations for animal welfare standards mean, for at least some practices, that they may diverge from those able to be provided by commercial farm in animal production–in other words, some improvements come at a cost. Who bears those costs, the methods of placing a value on them, and, ultimately, the behavior of consumers who pay for animal products in the market place, have been extensively described by, for example, McInerney (60, 61) and Norwood and Lusk (62)?

There are many insights of relevance to animal welfare. For example, the marketplace does not reflect the true values of society, only those things exchanged through markets. Nor can it include the views of people not involved in the marketplace–many of those active in advocating for better treatment of animals are, for example, vegans, or vegetarians. Market values can be distorted by subsidies and taxes, the lack of inclusion of externalities, like animal welfare (products from different systems are usually selected on the basis of cost and appearance rather than the impacts of those systems on the animals), and a lack of informed understanding of the differences between different production systems. Finally, consumers value products differently for different reasons and are free to alter their purchasing behavior. Therefore, it is usually necessary for state intervention to ensure efficiency and fairness, for example in setting minimum standards and in redistributing the costs and benefits.

This more involved understanding of economics reveals several interesting aspects about animal welfare. Firstly, food prices vary for all sorts of reasons (seasons, taxes, instore specials, supply) so perhaps the impacts of balancing animal welfare against food security and availability is over simplified. It is important to know the costs of improving animal welfare and how they might be distributed across the food supply chain, as well as the impacts on various consumers. Secondly, the market generally provides little or no reward or incentive to produce to higher animal welfare standards–such standards are increasingly seen as a cost of production, or of market access. As animal welfare is a value largely attributed to farmers providing good husbandry and stockmanship, not consumers, farmers may require economic signals and incentives to reflect the value consumers and society place on animal welfare. Finally, it is important not to overlook the complexity of the food supply system with its many opportunities for people to mistrust, exploit, distort, overlook, or remain wilfully blind to what occurs.

There are, then, many potential economic means of maintaining and enhancing animal welfare. Taxes and subsidies are one option and some countries link subsidies to successful animal welfare inspections. Another method is labeling products from preferred animal welfare-friendly systems enabling motivated consumers to support them. Tradable permits may enable those in more animal welfare-friendly systems to effectively subsidize less humane farming systems. Lastly, quotas can be used to ensure production is limited so that returns cover the expenses of favored systems.

What the above discussion highlights is that animal welfare is not just about the expectations society may have for how animals should be treated by the person in charge of them, but that it is part of a wider and more complex system. A common caricature of much public concern regarding poor animal welfare practices, is that it reflects an excessive focus on profits and uncaring individuals, for example, “farmers are a nasty, greedy, whinging lot.” While leaving aside the instances of animal neglect resulting from difficult personal circumstances, relationships and ill-health (18, 63, 64), and the fact that greed may play a part in some cases, such an understanding does not reflect the influence of economic factors. Animal welfare, McInerney (60) argues, is ultimately an economic or socio-political issue, a subset of human welfare. The place and role of economic understanding in animal welfare is yet to be fully realized.

## Ethics, Economics, and Pastoral Animal Welfare

Maintaining animals in suitable condition, expectations for the provision of shelter and a comfortable resting area free of mud, and the impacts of painful husbandry procedures, highlight the contested nature of pastoral animal welfare. The well-being of animals is compromised for human benefit, often expressed in economics for those involved in farming and its related industries, but also in the supply of safe and affordable food for others. Depending on individual and group perspectives, compromises are sometimes justified, sometimes not. It is society's consensus which ultimately decides when, for example, animals' needs can be legitimately thwarted, or those exposed to excessive mud or hot sun should have access to resources providing greater comfort and well-being. There are balances and limits in the use of animals–generally production is not maximized at the expense of welfare, and welfare is not maximized at the expense of production.

Poor animal welfare raises concerns and expectations amongst farmers, farm industries and the public alike. While, for example, the provision of shelter is part of good farming, there are different understandings of what is good, and different barriers or constraints to providing shelter, including finances, time and resources. Furthermore, there are different expectations of when shelter is required–for the comfort of the animal, that required to maintain its productivity, or that required to survive. Such differences also reflect different understandings of animal welfare–what the animal experiences, how it performs, whether it is natural and even if it is being treated with dignity and respect. Finally, there is the challenge of addressing something best expressed in the view “a cow on a hot day, yeah she's hot, we all get hot. Is that really a problem?” The contested degree of compromise to animals is crucial to determining what compromises animals can be expected to endure, and when assistance or resources should or must be provided, commensurate with public expectations, to assist them to cope.

To the above difficulties, we can further add the insights revealed by using body condition as a management tool, i.e., of the productivity of the herd or flock, and as an indicator of animal welfare, what the individual animal feels or experiences. For example, animals in good body condition traditionally may have been expected to endure muddy conditions. Similarly, the thwarting of animals' behavioral needs, like rooting in pigs, to prevent pasture and soil damage. These examples raise the issue of what needs are important–are some more important than others or, as legislation and the expectations of the five freedoms seem to imply, are all equal and thus must be given equal weight. Can adequate nutrition outweigh the discomfort of mud? And which human needs outweigh animals' needs? Does protecting the environment outweigh preventing pigs from rooting? One way worthy of further exploration is to perhaps weight animals' needs (65) such those sustaining life (e.g., food), health (e.g., shelter), and comfort (e.g., environmental complexity). This concept is akin to Abraham Maslow's well-known theory of human motivation and has been adapted to animal welfare (66). What it will require, however, is a greater understanding of the importance of social interactions and bonds which lessen individuals' demands for energy expenditure and skills, helping them to survive, reproduce, and care for their offspring, enhancing physical and mental health and thus well-being [see (67)]. The place of social behaviors is yet to be fully integrated into hierarchies of motivation.

Collectively, these aspects highlight the subjective and values-based complexity of animal welfare. Animal welfare, then, has been likened to a “wicked problem” (68)–difficult to describe, complex, changing and subject to inconsistencies and considerable debate. Wicked problems are not easily solved but are, at best, managed and progressed with understanding and compassion. Not surprisingly, there are many directives in guidelines, standards and regulations to provide such resources. Thus, while the resources the animal has access to are in the farmer's hands, expectations are increasingly influenced and determined by the wider community and societal expectations. What is not fully acknowledged is the influence, and indeed role, of that wider community and society, in helping maintain and enhance animal welfare other than in “telling” or “wanting” to “manage a farmer's resources at no cost or risk to themselves” (69).

Though the welfare of an animal is largely dependent on knowledge, beliefs and circumstances of the individuals caring for them, as noted animal welfare standards are determined by society. Consensus requires acknowledging the full spectrum of ethical and economic perspectives that provide opportunities and constraints for individual pastoral farmers. For instance, the recognition that economics is a key driver of farming systems is connected with the view that, at least some consumers see themselves as part of the problem. Society's role in pastoral farming welfare may be addressing the factors beyond the ability of farmers to control, such as financial interest and international exchange rates and consumer preferences, factors which affect the ability of those in charge of animals to care for them. A more sustainable vision of animal welfare borne of understanding the connection between animals and all people, may enable society to fairly balance the demands that livestock have with those of the wider community.

## Discussion

Land use has changed from the time of hunter-gatherers and nomadic pastoralists, to extensive and intensive settled pastoralism, and finally industrial or factory farming (70). This has shaped human activities from earliest times (71), enabling many people to live without being directly involved in raising and killing animals for food. These changes have undoubtedly contributed to the different values, preferences and prejudices people have toward the rightful place of animals in society. While the welfare of most animals reflects the care provided by those in charge of them, it is influenced by the wider community, and thus subject to contested scrutiny. The examples described in this chapter illustrate some of the contested issues in animal welfare, tensions borne of animals being compromised for human benefit, whether they are for reasons of financial costs, environmental degradation, or practicality.

Given the different viewpoints and ways of justifying stances, who should help decide society's stance on contested issues? Although there are many perspectives, discussion of three of those: scientists and veterinarians; citizens; and stockpeople, are the subject of this discussion because of the strong beliefs in their place, beliefs that may or may not withstand critical scrutiny. For instance, one perspective is that “there is no doubt that veterinarians are the best equipped and most committed profession to lead the community in animal welfare debate” (72). Such a view ignores the fact that animal welfare is a social construct and not exclusively scientific or veterinary. Furthermore, although veterinarians are well-trained in animal health, they are arguably less familiar with other aspects of animal welfare such as what the animal is experiencing (73), limitations which are now beginning to be more widely addressed in veterinary teaching curricula. Similarly, there are calls for science to guide socially contested issues “with rational application of sound scientific principles.” Such calls must be tempered by remembering, for example, that scientific advice need not be sound. For example, as recently as early last century survival rates of children in orphanages were terrible. At the time of the rising recognition of the value of cleanliness in preventing disease, and a desire to make the young field of psychology into a truly objective science, it led to a professional crusade by JB Watson, among others, against the evil of affection (“mother love is a dangerous instrument”). Such advice was given authority because its proponents were objective and scientific experts [see (74)]. In contrast [see (75)], the Scottish philosopher David Hume stated, “reason is the slave of passions and should be.” Furthermore, Enrich Fromm held that “reason flows from the blending of rational thought and feeling. If the two functions are torn apart, thinking deteriorates into schizoid intellectual activity and feeling deteriorates into neurotic life-damaging passions.” Thus, views based on selected or narrow professional understandings not only have a tendency to measure and value what can easily be assessed rather than what is important to the animal (76), they ignore the value of engaging with all our ways of knowing–ethics, common sense, intuition, imagination, memory, and reason (77).

Another perspective is that advanced by advocacy and activist NGO (non-governmental organizations) interests, often with or through the involvement of popular and social media. Although there are many different motives for an interest in animal welfare (78), including an identity for a minority, a means of expressing prejudiced views and class conflict, to be a part of more general protest movements, or even profit or enlightened self-interest, undoubtedly the main motive is a genuine concern for the well-being of animals. The growth of NGO interests may also reflect persons in charge and regulatory groups not doing their, or society's, job, or at least not engaging in the issue publicly, enabling “the most shrill and dramatic articulations” (79) that tend to demand attention, or those seeking “to privilege the transient urges of the mob over and above social order” (80). This is hardly a sound approach to addressing contested and complex issues. Debates about farming, including animal welfare, have been considered “disappointing intellectually, ethically, and politically.” Fraser (81) considers the debate has not resulted in genuine understanding of how farming affects animals, the environment, and the public; the polemic nature of many of the accounts has polarized the debate preventing critical analysis; and the debate has failed to produce shared understanding and consensus. In overcoming these short comings, those in society need to avoid simply aligning themselves with stances and seek knowledgeable analysis of the issues. Such an approach may require the creation of a forum enabling all parties to explore the various aspects of contentious issues (82). There is little time, resource or will to undertake such critical analysis when in the glare of social and public media frenzy.

Finally, we might ask if society, in determining animal welfare standards, is at risk of disregarding the invaluable perspectives of those husbanding animals since it is those persons in charge of animals who are arguably the most important determinants of their welfare. Stockmanship ensures animals have the resources they need to be comfortable, fit and feeling good. Kilgour and Dalton (83) included in the last chapter of their practical guide *Livestock Behavior* that there is the potential for better handling by allowing young animals to learn behaviors, such as lambs learning to eat drought feed whilst they are still with their dams, opportunities generally excluded by management practices such as early weaning and maintaining animals in age and sex cohorts. “Using the old to teach the young” may well apply to stockpeople, professionals and advocates, especially in the current era of narrow specialized expectations.

**Figure 3:**
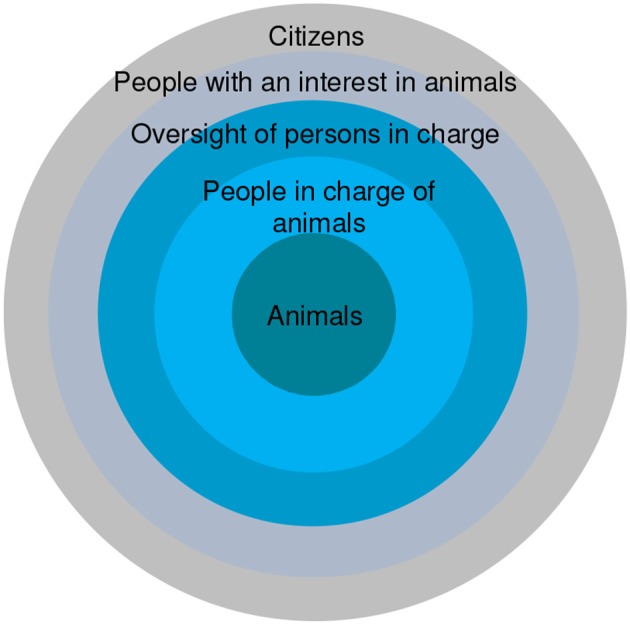
A schematic representation of the animal welfare system [from (85)].

These different perspectives suggest the issue is not so much who is best placed or qualified to determine animal welfare, but who brings knowledge, and practical experience, whether as a veterinarian, a scientist, a farmer or shepherd, or whatever. It is perhaps more relevant to think of animal welfare in terms of a system, since humans and animals are socially and ecologically interdependent (84). In one depiction (85) at the center of the system are animals. Then there are the persons in charge (e.g., farmers and farm workers); those with oversight of the persons in charge (e.g., animal welfare inspectors and regulatory advisory bodies); and those with an interest in animals (e.g., consumers of food, commerce interests, and animal advocates and activists). Finally, there are citizens, who, while not necessarily having direct vested interests in animals, have a special role in the democratic process. There are many examples of the individuals and groups in society making up the animal welfare system and they can be thought of as actors arranged in concentric bands (Figure 3).

Arranged in this way, the system acknowledges that each group has a role, and thus a responsibility, for animal welfare. Like tourists, individuals within each of the bands see the world from their own perspective in a varied but often limited way. Learning more of the features and expectations of others in different bands may act to change or reinforce our responses, in short having a genuine understanding of each other's interests and roles. It has been suggested that this is undertaken by identifying the issues, providing information and involving people (86, 87). In other words, taking responsibility for complexity by understanding animal welfare as a complex problem, recognizing that there are constraints and opportunities, giving people more autonomy by engaging local institutions, building trust with stakeholders, taking accountability for learning, and broadening dialogues (88, 89). In order to have good welfare, perhaps the most important thing is to give those in charge of animals the confidence, resources and opportunities to develop and deliver what they are best placed to do. Animal welfare is important but not all important–the environment and people, along with the outputs from farming (90) must also be placed within the context of the whole.

Farmers, like many others, are having to respond to a dynamic and complex world and conversations may be better managed as part of a wider debate on environmental management, markets and social expectations. In other words, animal welfare interests must be prepared to couch their preferences within the context of the farming system, and not just the experiences of the animal. It is suggested that society cannot merely tell farmers what to do any more than farmers can expect society to understand farmers' “reality.” The future may lie not so much in emphasizing productivity and profitability, but in understanding what animals are experiencing and in building better connections with people to produce more sustainable and equitable farming practices (90). It will be necessary to acknowledge the complexity of the issue borne of different animals, environments and people; and that initiatives may be better managed as part of wider expectations. The future may well-involve society moving from telling or expecting farmers to know how to manage their resources, to encouraging them by providing the confidence, resources and opportunities to provide those resources.

Part of managing expectations may require engagement with the wider community–mediating between the reality of animal needs and the demands of public perception, whether informed or uninformed.

## Author Contributions

The author confirms being the sole contributor of this work and has approved it for publication.

## Conflict of Interest

The author declares that the research was conducted in the absence of any commercial or financial relationships that could be construed as a potential conflict of interest.
